# Co-infection of SARS-CoV-2 with other viral respiratory pathogens in Yogyakarta, Indonesia: A cross-sectional study

**DOI:** 10.1016/j.amsu.2022.103676

**Published:** 2022-04-30

**Authors:** Eggi Arguni, Endah Supriyati, Mohamad Saifudin Hakim, Edwin Widyanto Daniwijaya, Firdian Makrufardi, Ayu Rahayu, Anwar Rovik, Utari Saraswati, Farida Nur Oktoviani, Nenes Prastiwi, Titik Nuryastuti, Tri Wibawa, Sofia Mubarika Haryana

**Affiliations:** a Department of Child Health, Faculty of Medicine, Public Health and Nursing, Universitas Gadjah Mada, Sekip, Sleman, Yogyakarta, 55281, Indonesia; b Centre for Tropical Medicine, Faculty of Medicine, Public Health and Nursing, Universitas Gadjah Mada, Sekip, Sleman, Yogyakarta, 55281, Indonesia; c COVID Laboratory, Faculty of Medicine, Faculty of Medicine, Public Health and Nursing, Universitas Gadjah Mada, Sekip, Sleman, Yogyakarta, 55281, Indonesia; d Department of Microbiology, Faculty of Medicine, Public Health and Nursing, Universitas Gadjah Mada, Sekip, Sleman, Yogyakarta, 55281, Indonesia; e Department of Histology and Cell Biology, Faculty of Medicine, Public Health, and Nursing, Universitas Gadjah Mada, Sekip, Sleman, Yogyakarta, 55281, Indonesia

**Keywords:** COVID-19, SARS-CoV-2, Co-infection, Viral respiratory pathogens, Coronavirus

## Abstract

**Background:**

Growing evidence shows that viral co-infection is found repeatedly in patients with Coronavirus Disease–2019 (COVID-19). This is the first report of SARS-CoV-2 co-infection with viral respiratory pathogens in Indonesia.

**Methods:**

Over a one month period of April to May 2020, SARS-CoV-2 positive nasopharyngeal swabs in our COVID-19 referral laboratory in Yogyakarta, Indonesia, were tested for viral respiratory pathogens by real-time, reverse transcription polymerase chain reaction (RT-PCR). Proportion of co-infection reported in percentage.

**Results:**

Fifty-nine samples were positive for other viral respiratory pathogens among a total of 125 samples. Influenza A virus was detected in 32 samples, Influenza B in 16 samples, Human metapneumovirus in 1 sample, and adenovirus in 10 samples. We did not detect any co-infection with respiratory syncytial virus. Nine (7.2%) patients had co-infection with more than two viruses.

**Conclusion:**

Viral co-infection with SARS-CoV-2 is common. These results will provide a helpful reference for diagnosis and clinical treatment of patients with COVID-19.

## Highlights

• Viral co-infection with SARS-CoV-2 is common.
• Co-infection by two viral respiratory pathogens is prevalent.
• Pandemic transmission control may also impact in other viral respiratory infections.
• Early identification of co-infection is necessary, given differences in treatment.
• Simple laboratory diagnosis algorithm may be applied to screen viral co-infection.

## 1 Introduction

Since December 2019, the world has been dealing with a Severe Acute Respiratory Syndrome Coronavirus 2 (SARS-CoV-2) outbreak, which has had a devastating effect on public health as well as becoming a significant social and global economic burden. Indonesia is one of the countries most affected by Coronavirus Disease-2019 (COVID-19) with over 1.8 million confirmed cases and nearly 51,000 deaths as of June 3, 2021 (1). Yogyakarta is a remote province on the island of Java with a relatively high population density. During the same time period, 45,400 cases and 1200 deaths were reported in Yogyakarta [1].

In contrast to the first report in the early phase of the pandemic in Wuhan, China, which reported that co-infections with other respiratory pathogens in COVID-19 patients were infrequent [2], a study conducted in Northern California, USA revealed that the rates of viral respiratory co-infections had risen up to 21% [3].

Since SARS-CoV-2 have several similarities with common viral respiratory pathogens in terms of clinical presentation and modes of transmission, it is possible for co-infection to occur between SARS-CoV-2 and other viral respiratory pathogens. Recent studies showed that such co-infections may also aggravate the patients’ clinical condition [[4], [5], [6], [7]]. The concerns of health authorities worldwide were directed toward the burden of these concomitant infections during the initial pandemic months and many clinical guidelines reflected this by indicating both prevention of human respiratory syncytial virus (hRSV) and treatment of influenza for suspected cases [8].

Up to this moment, there is a lack of data regarding co-infection with other viral respiratory pathogens in patients with confirmed COVID-19 in Indonesia. In this brief report, we examine the frequency of respiratory viral co-infection among 125 SARS-CoV-2 positive nasopharyngeal swab samples at one of the COVID-19 referral laboratories located in Yogyakarta, Indonesia.

## 2 Methods

Positive respiratory specimens (nasopharyngeal swabs) of SARS-CoV-2 tested between April to May 2020 at the COVID Laboratory of the Faculty of Medicine, Public Health and Nursing, Universitas Gadjah Mada Yogyakarta were screened for other viral pathogens: human Influenza A and B viruses, hRSV, Human metapneumovirus (hMPV) and Adenovirus. Ethical approval was received from the Medical and Health Research Ethics Committee (KE/FK/0660/EC/2020). All methods for detection of SARS-CoV-2 with reverse transcription-polymerase chain reaction (RT-PCR) were performed correspondingly to the relevant guidelines. Proportion of specimens positive for SARS-CoV-2 and for each non-SARS-CoV-2 pathogen were identified. Data are presented with age group of patients and the proportion of gender. RNA from the nasopharyngeal swab samples of the patients with confirmed COVID-19 were used as a template for real-time RT-PCR to detect the presence of other viral respiratory pathogens. The PCR mixture contained 2 μl of forward and reverse primers (final concentration of 10 μM), 10 μl of 2X Sensifast Sybr No Rox (Bioline, USA), 0.2 μl of Reverse Transcriptase, 0.4 μl of Rnase Inhibitor, 3.4 μl of PCR grade water, and 4 μl of RNA sample. Amplification conditions were set-up as follows: reverse transcription (RT) step at 45 °C for 10 min; initial denaturation at 95 °C for 2 min, followed by 40 cycles of 95 °C for 5 s, 60 °C for 10 s, and 72 °C for 5 s. The primers used in this study have been described previously (Table 1) [9]. This study has been reported in line with the Strengthening the Reporting of Cohort Studies in Surgery (STROCSS) criteria [10].

**Table 1:** List of primers for real-time RT-PCR.

Target	Primer name	Sequence (5′-3′)	Gene Target
Influenza A	InflA_F	GAC AAG ACC AAT CCT GTC ACY TCT G	M gene
	InflA_R	AAG CGT CTA CGC TGC AGT CC	
Influenza B	InflB_F	TCG CTG TTT GGA GAC ACA AT	M gene
	InflB_R	TTC TTT CCC ACC GAA CCA	
Respiratory Syncytial Virus A, B	RSV_F	ATG AAC AGT TTA ACA TTA CCA AGT	F gene
	RSV_R	GTT TTG CCA TAG CAT GAC AC	
Adenovirus (Resp)	AdvResp_F	CAG GAC GCC TCG GRG TAY CTS AG	Hexon gene
	AdvResp_R	GGA GCC ACV GTG GGR TT	
Human Metapneumovirus	hMPV_F	AGC TTC AGT CAA TTC AAC AGA AG	F gene
	hMPV_R	CCT GCA GAT GTY GGC ATG T	

## 3 Results

As shown in Table 2, our study detected viral respiratory pathogens in 59 of 125 samples (47.2%). We detected co-infection with Influenza A virus in 32 samples, Influenza B in 16 samples, hMPV in 1 sample, and adenovirus in 10 samples. We did not detect any co-infection with hRSV. Notably, 9 (7.2%) patients had co-infection with more than two viruses (Table 3).

**Table 2:** Co-infection of SARS-CoV-2 with other viral respiratory pathogens.

Parameter		N TOTAL	Inf-A Result	Inf-B Result	hMPV Result	hRSV Result	Adeno Result
Age	1 < y.o < 5	2	0	0	0	0	0
	5 < y.o < 18	6	3	1	1	0	1
	18 < y.o < 45	46	13	3	0	0	4
	45 < y.o < 60	41	11	5	0	0	5
	≥60	30	5	7	0	0	0
	Total	125	32	16	1	0	10
Sex	Male	59	15	3	0	0	2
	Female	66	17	13	1	0	8
	Total	125	32	16	1	0	10

**Table 3:** Multivirus-infection.

Samples	Age (year)	Viral respiratory pathogen
#33	57	SARS-CoV-2 + Influenza A + Adenovirus
#51	46	SARS-CoV-2 + Influenza A + Adenovirus
#54	15	SARS-CoV-2 + Influenza A + Adenovirus
#61	57	SARS-CoV-2 + Influenza A + Adenovirus
#79	57	SARS-CoV-2 + Influenza A + Adenovirus
#83	19	SARS-CoV-2 + Influenza A + Influenza B + Adenovirus
#116	37	SARS-CoV-2 + Influenza A + Adenovirus
#121	71	SARS-CoV-2 + Influenza A + Influenza B
#122	51	SARS-CoV-2 + Influenza A + Adenovirus

## 4 Discussion

There is existing evidence of pathogenic competition between respiratory viruses, including between human influenza viruses and seasonal human coronaviruses (OC43, HKU1, NL63, and 229E) [[11], [12], [13]]. Therefore, even in a pandemic situation, several viral respiratory pathogens should be considered, when establishing the initial etiology and appropriate treatment. At the population level, simultaneous detection of respiratory viruses, such as influenza and SARS-CoV-2, can be employed as an early prediction model of future outbreaks of both viruses.

Our study showed a higher prevalence of viral co-infection (47.2%) compared to the prevalence of 31.5% reported in Jiangsu Province, China [14]. Additionally, the prevalence was much higher compared to those reported in Northern California (21%) [3], Australia (8%) [15], Turkey (2%) [16], and Singapore (1.4%) [17]. The latest systematic review estimated that 3% of hospitalized patients with confirmed COVID-19 were also co-infected with another respiratory virus, mostly hRSV and human influenza A virus [18]. By using a test negative design, one study indicated that the risk of testing positive for SARS-CoV-2 was significantly lower among influenza positive cases [7]. Another study reported that <3% of those testing positive for SARS-CoV-2 had co-infection with Influenza A virus. This frequency was lower in SARS-CoV-2 negative patients in which 13% were influenza positive [19]. A similar interaction happens between influenza and other viruses, such as seasonal coronaviruses and rhinovirus [[11], [12], [13],^20^]. SARS-CoV-2 apparently has a slower growth rate than influenza virus. The growth of SARS-CoV-2 is suppressed by the influenza virus if they both simultaneously infect the host. Co-infection between these two viruses would be more readily detected, if influenza virus infection followed SARS-CoV-2 infection [21].

This phenomenon suggests a pathogenic competition between those respiratory tract-infecting viruses. Furthermore, it is hypothesized that general and non-specific immune responses (such as interferon) against the first viral infection (i.e., influenza virus), may help inhibiting the secondary SARS-CoV-2 infection [22]. The increased risk of severe disease and mortality were also found to be associated with co-infection between SARS-CoV-2 and Influenza A virus, even though this appears to not be correlated to the independent effects of each virus [18].

Recent research in Indonesia reported the prevalence of influenza within severe acute respiratory infection (SARI) patients was 12.1% [23]. Even though the surveillance data for the seasonal incidence of influenza virus infection are lacking in Indonesia, influenza viruses and SARS-CoV-2 share the common route of transmission and similar clinical manifestations, during this pandemic, co-infection with these viruses should be carefully considered throughout the year.

In this study, we did not identify co-infection with hRSV, similar to another study in Brazil. That study did not find any co-infection with hRSV in the region which has a very well-defined and significant annual hRSV and influenza transmission [24]. This finding, again, alerts us to the hypothesis of a possible pattern of competition among respiratory viruses, but the public awareness of SARS-CoV-2 pandemic for transmission control such as using face mask, local social distancing, working from home, and the closing of schools and daycare centers can be associated with these observations.

hMPV co-infection was detected in only 1 sample (1/125). Previously, identification of co-circulation of hMPV and SARS-associated coronavirus during a nosocomial SARS-CoV outbreak raised the possibility of significant interaction [25]. hMPV transmission was reported as a serious outbreak in health care facilities [26]. Furthermore, this current study revealed the proportion of Adenovirus co-infection was around 8% of tested samples, which was similar with the pool proportion of Adenovirus co-infection in total viral detections reported in a systematic review [18].

Co-infection by two viral respiratory pathogens is prevalent, given similar routes and modes of transmission [27,28]. Nevertheless, early identification of coinfection is necessary given the differences in treatment and prognosis. Antiviral therapy is currently available for influenza virus infection (eg oseltamivir) as well as unlabeled experimental drugs (eg lopinavir/ritonavir and hydroxychloroquine) are becoming common practice in the treatment of COVID-19.

We acknowledge that our study was not without some limitations. All specimens tested in this study were only the SARS-CoV-2 positive samples with the result that we could not reveal the proportion of other viral pathogens in SARS-CoV-2 negative samples. Second, we could not analyze detailed information about epidemiology and clinical manifestations of patients since some information was not available in our laboratory. Third, all specimens tested for these other viral co-infections were from the second frozen-thawed samples, which could contribute to the failure to identify the viruses that originally may have been present at very low titer. Finally, we could not identify the sequential course of primary and secondary infections, so we are unable to explain the possible interaction.

Based on the result of this current study and similar other studies, the respiratory virus co-infections are more likely to occur during pandemic. The evident increase in risk among patients with co-infection has implications to support vaccination, not only for SARS-CoV-2 but also for influenza viruses. Simple laboratory diagnosis algorithm may be applied to screen viral co infection and the testing for influenza viruses is important in hospital inpatients with COVID-19 to identify those who might have different responses to antiviral therapy.

## 5 Conclusion

In conclusion, an early and rapid identification of concomitant viral respiratory pathogens is important to improve diagnosis, clinical management and patients’ prognosis. Further research is required to better understand the pathogenic role of viral coinfection in respiratory disease.

## Provenance and peer review

Not commissioned, externally peer reviewed.

## Sources of funding

This study was funded by COVID Pemandatan UGM .

## Ethical approval

This study has been approved by the Ethical Committee of Faculty of Medicine, Public Health and Nursing, Universitas Gadjah Mada/Dr. Sardjito Hospital (KE/FK/0660/EC/2020).

## Consent

Written informed consent was obtained from the patient for publication of this study and accompanying images. A copy of the written consent is available for review by the Editor-in-Chief of this journal on request.

## Author contribution

Eggi Arguni and Endah Supriyati contributed equally to this work. All authors conceived the study, drafted and critically revised the manuscript for important intellectual content. Firdian Makrufardi critically revised the manuscript for important intellectual content. All authors read and approved the final draft. All authors facilitated all project-related tasks.

## Registration of Research Studies

Research Repository Faculty of Medicine, Public Health and Nursing, Universitas Gadjah Mada

Register Unique Identifying Number (UIN): 202202115

## Guarantor

Eggi Arguni.

## Declaration of competing interest

No potential conflict of interest relevant to this article was reported.
